# Tobacco control: Developing an innovative and effective global strategy

**DOI:** 10.1371/journal.pmed.1002308

**Published:** 2017-05-30

**Authors:** The PLOS Medicine Editors

**Affiliations:** Public Library of Science, San Francisco, California, United States of America and Cambridge, United Kingdom

## Abstract

In this month’s editorial, the *PLOS Medicine* Editors discuss the campaign for World No Tobacco Day 2017.

In 2014 through 2015, an Ebola outbreak in West Africa claimed more than 11,000 lives and caused very substantial burdens of ill health, societal disruption, and fear [1]. The uncertain response to this unexpected outbreak, together with concerns about prospects for the disease to spread between continents with catastrophic consequences, led to a crisis of existential proportions for WHO and its Director-General. Since then, efforts have continued towards understanding the key characteristics of the outbreak and their causes, and seeking to acquire and use scientific knowledge to prepare for future events. Yet, imagine, for a moment, a hypothetical infectious disease that contributes, very predictably, to some 6 million deaths per year around the world. The full health burden of the disease is even greater and is growing alarmingly. Its main causal exposure often begins in adolescence and is sustained over the long term by addictive properties, reinforced by cultural associations with carriers’ status and confidence. Serious and often incurable health consequences are highly likely, usually decades later. Though not infectious in the conventional sense, of course, this ailment—tobacco use in its different guises—is, unfortunately, very far from hypothetical.

World No Tobacco Day falls on May 31, 2017, and this year has the plausible theme “Tobacco—a threat to development” [2]. The WHO campaign aptly notes the disastrous and all too well-known health effects of tobacco smoking, with the burden expected to escalate to an appalling 8 million deaths per year by 2030, owing to growth in low- and middle-income countries. Avoidable morbidity from associated cardiovascular diseases, chronic obstructive pulmonary disease, cancers, and other illnesses will also grow substantially, with the poorest population groups likely to suffer disproportionately. In the diffuse nomenclature of the United Nations Sustainable Development Goals (SDGs), efforts to achieve a decrease in tobacco use should be driven by the aim of a one-third reduction in premature deaths from noncommunicable diseases, which is specified in target 3.4 of the 17 overall goals to be reached globally by 2030.

Viewing tobacco use in the context of development allows the campaign to highlight the environmental impact of tobacco farming, including an effect on deforestation that is projected to be substantial. Tobacco-dependent demand for healthcare and services and the decreased productivity caused by ill health concentrated in deprived sectors of societies can also be expected to exert important negative effects on development. Increasing tobacco taxes is cited by WHO as one important remedial factor, with the potential to raise US$190 billion “for development.” As an example of the possible benefits of taxation, the number of cigarettes sold in China was reported to have fallen by 3.3% in 2015 to 2016, a time during which the tax rate on cigarettes was raised from 5% to 11% [3]. There was also a greater decrease in the sales of cheaper cigarettes, suggesting potential targeted benefit for young and low-income smokers.

In the context of economics and development, tobacco poses an especially thorny problem. There is no doubt that global tobacco businesses are responsible for making and promoting a product with extremely damaging long-term effects on health and that these effects have been downplayed disingenuously for decades. It is encouraging to see the WHO campaign noting that “an increasing number of countries are creating firewalls to ward off interference from the tobacco industry in government tobacco control policy.” However, the perverse discipline of tobacconomics invites skepticism. China, for example, has more than 300 million smokers, with the health of many more people likely to be damaged by the effects of secondhand smoke. The near-monopoly of China’s state tobacco manufacturer has been estimated to contribute about 7% of government revenues [4], which has surely contributed to the country’s development and economic growth. Despite the promise of taxation and other population-level approaches for reduction of the health burden of tobacco use [5], the damaging symbiotic relationship between tobacco use and economic growth could constrain implementation of smoking cessation strategies in the countries in which they are most needed.

WHO’s next Director-General, whose identity was unknown at the time of writing, is expected to be appointed at the World Health Assembly in May of this year. The successful candidate will have a demanding to-do list [6], but one of his or her highest priorities should be to bypass the complexity of the SDGs and take decisive action against the world’s pandemic of tobacco use. WHO has a noteworthy track record on the issue, with the 2005 Framework Convention on Tobacco Control having led the international public health agenda towards action on tobacco, including the imposition of taxes and other measures to reduce demand, bans on advertising, and the provision of smoking cessation programs (observation of the treaty is included as SDG target 3.A, for “strengthened implementation…as appropriate”). However, the grim facts on smoking incidence and prevalence, and on the grave and growing health consequences, call strongly for renewed action against tobacco. As sponsor of the “Ebola ça suffit!” ring-vaccination trial [7], WHO has recently shown its ability to take the lead in developing scientific solutions to important health threats. Because conflicts of interest in the funding and process of scientific studies are so common in the domain of tobacco and smoking, active involvement by WHO could have special value in this area. In addition to taking urgent steps to reenergize the public health trajectory in low- and middle-income countries to improve tobacco control, providing new scientific leadership on the issue—whether towards novel behavioral approaches, smoking cessation interventions, or harm reduction—should also be a priority for the agency.
